# The repeatable opportunity for selection differs between pre‐ and postcopulatory fitness components

**DOI:** 10.1002/evl3.210

**Published:** 2020-12-25

**Authors:** Lucas Marie‐Orleach, Nikolas Vellnow, Lukas Schärer

**Affiliations:** ^1^ Department of Environmental Sciences, Zoological Institute University of Basel Basel 4051 Switzerland; ^2^ School of Biology, Centre for Biological Diversity University of St Andrews St Andrews KY16 9TH United Kingdom; ^3^ Natural History Museum University of Oslo Oslo 0318 Norway; ^4^ CNRS, ECOBIO (Écosystèmes, Biodiversité, Évolution) – UMR 6553 Université de Rennes 1 Rennes 35000 France; ^5^ Evolutionary Biology Bielefeld University Bielefeld DE‐33615 Germany

**Keywords:** Cryptic female choice, hermaphrodites, mate choice, measuring selection, opportunity for sexual selection, sperm competition

## Abstract

In species with multiple mating, intense sexual selection may occur both before and after copulation. However, comparing the strength of pre‐ and postcopulatory selection is challenging, because (i) postcopulatory processes are generally difficult to observe and (ii) the often‐used opportunity for selection (*I*) metric contains both deterministic and stochastic components. Here, we quantified pre‐ and postcopulatory male fitness components of the simultaneously hermaphroditic flatworm, *Macrostomum lignano*. We did this by tracking fluorescent sperm—using transgenics—through the transparent body of sperm recipients, enabling to observe postcopulatory processes *in vivo*. Moreover, we sequentially exposed focal worms to three independent mating groups, and in each assessed their mating success, sperm‐transfer efficiency, sperm fertilizing efficiency, and partner fecundity. Based on these multiple measures, we could, for each fitness component, combine the variance (*I*) with the repeatability (*R*) in individual success to assess the amount of repeatable variance in individual success—a measure we call the repeatable opportunity for selection (*I_R_*). We found higher repeatable opportunity for selection in sperm‐transfer efficiency and sperm fertilizing efficiency compared to mating success, which clearly suggests that postcopulatory selection is stronger than precopulatory selection. Our study demonstrates that the opportunity for selection contains a repeatable deterministic component, which can be assessed and disentangled from the often large stochastic component, to provide a better estimate of the strength of selection.

**Impact Summary**

In many animals, the number of offspring an individual produces can depend on events occurring before, during, and after sexual reproduction. Traits allowing individuals to exclude same‐sex competitors, or to be preferentially chosen for reproduction by the opposite sex, provide advantages that can lead to the evolution of so‐called sexually selected traits, such as the red deer's impressive antlers or the peacock's colorful train. Although sexual selection was initially thought to only occur before copulation, intense—and often cryptic—periods of sexual selection may occur also after copulation, inside the female reproductive tract. However, understanding the relative importance of this pre‐ versus postcopulatory sexual selection is challenging because (i) internal postcopulatory processes are often difficult to observe, because females are usually not see‐through, and (ii) some individuals may be more or less successful just by chance. Here, by using genetically modified flatworms of the species *Macrostomum lignano* producing green fluorescent sperm, we could track donated sperm inside the highly transparent bodies of living sperm‐receiving worms. We exposed focal worms to three independent mating groups, in which we then (i) observed the mating interactions, (ii) tracked sperm in the focals' partners, and (iii) assigned paternity of their offspring to the focal worm. This allowed us to assess how good each focal was at mating, transferring sperm, and fertilizing the eggs of its partners. In each of these fitness components, we could assess the strength of selection, while excluding the effects of chance through a measure that we call the “repeatable opportunity for selection,” which includes information about how consistently a focal performed over its three mating groups. Our study suggests that selection is stronger on sperm‐transfer efficiency and sperm fertilizing efficiency than on mating success, which demonstrates that, although cryptic, sexual selection after copulation may be stronger than before copulation.

Sexual selection is a widespread evolutionary force that can promote the exaggeration of traits mediating reproductive success and can affect macroevolutionary processes, such as speciation and extinction (Lande 1981; Andersson 1994; Birkhead and Møller 1998; Kokko and Brooks 2003; Birkhead et al. 2009). It has long been accepted that competition for mate acquisition—either through competition among individuals of the same sex or through attraction of individuals of the opposite sex—may lead to the evolution of sex‐specific traits that cannot be explained by natural selection alone (Darwin 1859, 1871). Only later was it realized that sexual selection may also continue after mating: when females mate with multiple males, sperm of different males may compete for fertilization (sperm competition) and females may bias fertilization toward certain males (cryptic female choice) (Parker 1970, 1998; Charnov 1979; Thornhill 1983; Eberhard 1996). Modern views now consider that sexual selection often acts over multiple consecutive pre‐ and postcopulatory episodes of selection (Jennions and Kokko 2010; Shuker 2014). For instance, high reproductive success may entail high success in fighting with same‐sex individuals, attracting and copulating with multiple mating partners, successfully transferring or receiving optimal amounts sperm, and/or optimizing fertilization success. However, the generally cryptic nature of postcopulatory processes and the challenges associated with measuring sexual selection currently limit our understanding of how sexual selection operates in these pre‐ and postcopulatory episodes (Evans and Garcia‐Gonzalez 2016).

Specifically, although pre‐ and postcopulatory episodes of selection may constitute important fitness components, their quantification is often complicated by the challenging task of measuring sexual selection (Wade and Shuster 2005; Snyder and Gowaty 2007; Klug et al. 2010; Krakauer et al. 2011; Jennions et al. 2012; Henshaw et al. 2016). A common way to measure selection is through the relationship between certain traits expressed by the individuals and their fitness (e.g., selection gradients), which can be decomposed into pre‐ and postcopulatory fitness components (Lande and Arnold 1983; Arnold and Wade 1984; Henshaw et al. 2018). However, such a trait‐based approach is only indicative of the selection acting on the specific measured traits, and is thus not suitable to quantify and compare the total strength arising from different fitness components, because even in well‐studied organisms measurements of some traits important for fitness may always be missing. An alternative approach, which we hereafter call variance‐based, attempts to estimate the strength of selection through the variance in individual success (Bateman 1948; Wade 1979; Arnold and Wade 1984; Krakauer et al. 2011), positing that high variance in individual success can serve as an indicator for intense selection. However, the variance in individual success only sets the upper bound for selection on phenotypic traits (Crow 1958; Jones 2009). It is therefore indicative only of the “opportunity for selection” on traits, but not necessarily the realized selection, because some of the observed variance may result from stochastic events that are unlinked to specific phenotypic traits of the studied individuals. Nonetheless, the variance in mating success (opportunity for sexual selection) and in reproductive success (opportunity for selection ) are widely used metrics, and have permitted meaningful comparisons of the strength of sexual selection between the sexes (Fritzsche and Arnqvist 2013) or among species (Janicke et al. 2016).

The variance‐based approach includes the partitioning of the overall opportunity for selection into multiplicative fitness components (Arnold and Wade 1984), allowing the estimation of the opportunity for selection arising from different pre‐ and postcopulatory fitness components (Collet et al. 2012; Pélissié et al. 2012, 2014; Rose et al. 2013; Devigili et al. 2015; Janicke et al. 2015; Turnell and Shaw 2015; Marie‐Orleach et al. 2016). This body of work indicates that postcopulatory fitness components can in some cases explain large portions of the variance in male reproductive success, sometimes even exceeding those of precopulatory components (Evans and Garcia‐Gonzalez 2016). However, the variance‐based approach is not informative about the specific traits causing variance in individual success. Specifically, variance in individual success may be caused by deterministic factors linked to specific phenotypic traits on which selection can act (provided that they show underlying additive genetic variation). Such traits may include, for example, large armaments that increase success under fights or large testes that permit to succeed in sperm competition. Alternatively, variance may arise from stochastic events that are unlinked to specific traits of the individual, so that selection cannot actually act (although genetic drift might still be affected by the magnitude of variance). For example, an individual may happen to avoid exposure to a parasite out of pure luck and thus live to mate another day or it may just happen to be the first to encounter a female in a species with first‐male sperm precedence. Because the variance‐based approach indiscriminately lumps such deterministic and stochastic components together, some authors consider the variance‐based approach to be misleading (Klug et al. 2010; Jennions et al. 2012), which emphasizes that considerable caution is required when interpreting variance‐based metrics.

Here, we employed a variance‐based approach to measure the opportunity for selection arising from pre‐ and postcopulatory components of male fitness, while aiming to disentangle deterministic from stochastic sources of variance. For this, we built upon an approach presented in a previous study (Marie‐Orleach et al. 2016), which permits to decompose variance in male reproductive success into several fitness components (see below). In addition, for each fitness component, we also estimated the repeatability of individual success by sequentially exposing the same focal sperm donors (hereafter called focals) to multiple independent mating groups. This allowed us to estimate whether focals performed consistently across the mating groups or whether their performance varied greatly between these groups, which in turn permitted us to determine how much of the variance in their success was likely due to deterministic factors versus stochastic events. This additional repeatability assay permits weighting the variance observed in each fitness component (*I*) by its corresponding repeatability (*R*). We could thereby distinguish the portion of variance arising from each fitness component that is likely caused by deterministic factors—the repeatable opportunity for selection, *I_R_*—from the stochastic portion of the variance that is probably unlinked to any deterministic factors. This important distinction addresses a key criticism of the variance‐based approach (Klug et al. 2010; Jennions et al. 2012).

To this aim, we took advantage of features of the free‐living flatworm *Macrostomum lignano*—a simultaneous hermaphrodite that has become a powerful model system for research on sexual selection (Schärer et al. 2011; Janicke et al. 2013; Ramm et al. 2019; Patlar et al. 2020)—allowing us to quantify pre‐ and postcopulatory male fitness components (Fig. 1). Specifically, we sequentially exposed focals to three independent mating groups, and assessed, in each group, the individual male reproductive success of the focal in four multiplicative fitness components (as defined in Marie‐Orleach et al. 2016, while employing a nearly threefold larger sample size to improve parameter estimation). Briefly, we decomposed focal male reproductive success into (1) partner fecundity (i.e., the total number of offspring produced by all potential partners through their female sex function), (2) mating success (i.e., the proportion of all copulations in which the focal was involved), (3) sperm‐transfer efficiency (i.e., the proportion of sperm successfully received from the focal by all potential partners, given the focal mating success), and (4) sperm fertilizing efficiency (i.e., the proportion of offspring sired by the focal, given the proportion of focal sperm received) (see Fig. 1 and Methods for more details).

**Figure 1:**
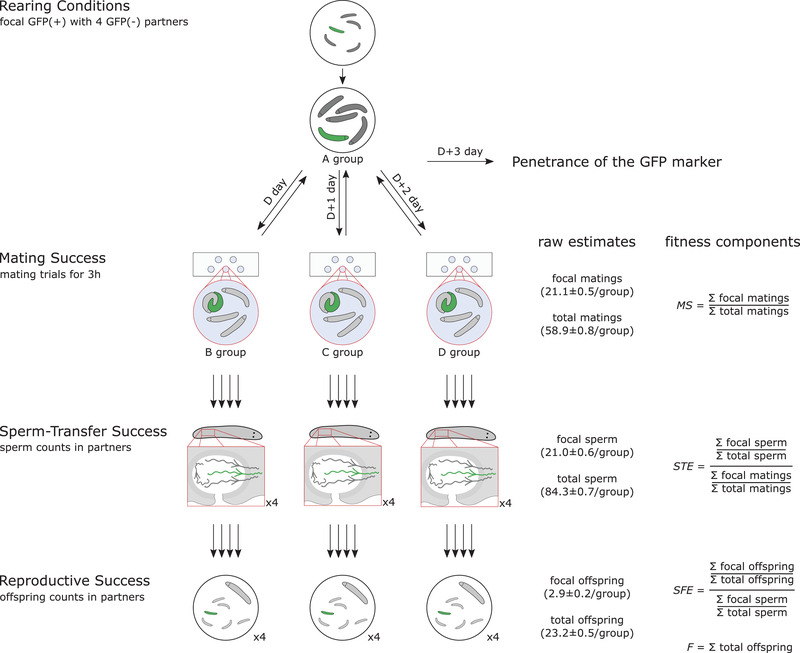
**Overview of the experimental design used to measure four multiplicative fitness components of male reproductive success, namely, mating success (*MS*), sperm‐transfer efficiency (*STE*), sperm fertilizing efficiency (*SFE*), and partner fecundity (*F*), derived from raw estimates obtained along different episodes**. GFP(+) focal worms were raised to adulthood from the juvenile stage together with four GFP(–) worms (their A group), and then sequentially exposed in 3‐h mating trials to three independent groups of four GFP(–) partner worms (their B, C, and D groups) over three consecutive days (and in between placed back into their A group). The multiplicative fitness components are derived from raw estimates (given as Mean ± SE in brackets) of mating success, sperm‐transfer success (sperm counts in the sperm‐storage organ of the partners, the female antrum), and reproductive success (offspring counts in isolated partners). Finally, after all the mating trials the focals were used to assess the penetrance of the GFP marker. Drawings are not to scale. Note that all fitness estimates were relativized for subsequent analysis (so that the means equal 1), which is denoted by an asterisk (i.e., mRS*, F*, MS*, STE*, and SFE*). See Methods for details.

We can estimate the last two (postcopulatory) fitness components because we can perform *in vivo* sperm tracking in *M. lignano* (Janicke et al. 2013; Marie‐Orleach et al. 2014; Wudarski et al. 2017). Briefly, by using transgenic focals that express green fluorescent protein (GFP) in all cell types, including the sperm cells, one can easily distinguish sperm cells donated by GFP(–) wild‐type competitors from those donated by the GFP(+) focals, because sperm can be observed directly inside the female reproductive tract of living partner worms in this highly transparent species. This provides powerful opportunities to quantify fitness components that are usually difficult to observe and cryptic, such as the number of sperm cells that a GFP(+) focal individual has successfully transferred to its partners after mating in groups (Janicke et al. 2013; Marie‐Orleach et al. 2016). Moreover, because counting sperm cells inside the female reproductive tract is noninvasive, and because the GFP marker is inherited by the offspring in a dominant fashion, paternity analyses of the GFP(+) focal can also easily be performed in the resulting offspring (Marie‐Orleach et al. 2014, 2016).

Our study demonstrates that the often‐used opportunity for selection (*I*) includes a large stochastic component, and that different fitness components are more or less affected by such a stochasticity. After excluding the stochastic component, we found that the deterministic component—the repeatable opportunity for selection (*I_R_*)—was significant in mating success, sperm‐transfer success, and sperm fertilizing success, but not in partner fecundity. Importantly, we found that the repeatable opportunity for selection is more than three times larger in sperm‐transfer efficiency and sperm fertilizing efficiency compared to mating success, which suggests that selection is more intense on postcopulatory episodes of selection compared to the precopulatory episodes.

## Methods

### MODEL ORGANISM

*Macrostomum lignano* (Macrostomorpha, Platyhelminthes) is a free‐living flatworm living in the upper intertidal zone of the Northern Adriatic Sea and the Eastern Mediterranean basin (Ladurner et al. 2005; Schärer et al. 2020). Laboratory cultures are fed with the diatom *Nitzschia curvilineata* and kept at 20°C in glass Petri dishes containing f/2 medium (Andersen et al. 2005). *Macrostomum lignano* is an outcrossing simultaneous hermaphrodite (Schärer and Ladurner 2003), showing multiple mating and high copulation rates (Schärer et al. 2004; Janicke and Schärer 2009). Copulation consists of the reciprocal intromission of the male copulatory organ (called stylet) into the partner's female sperm‐receiving and sperm‐storage organ (called female antrum) (Schärer et al. 2004; Vizoso et al. 2010). Worms are transparent, which allows us to observe and measure numerous internal structures *in vivo*, including the number of received sperm cells stored inside the female antrum (Janicke et al. 2011; Marie‐Orleach et al. 2016).

The recently established *M. lignano* transgenic lines expressing GFP (Janicke et al. 2013; Marie‐Orleach et al. 2014; Wudarski et al. 2017) offer two critical features for this study. First, GFP expression is ubiquitous, which means that GFP(+) individuals produce and transfer GFP(+) sperm cells that can be observed—and visually distinguished from GFP(–) sperm cells—*in vivo* inside the female antrum of sperm recipients (Janicke et al. 2013; Marie‐Orleach et al. 2014). Second, because the GFP marker is dominant, one can use it to efficiently assess parentage in offspring, by checking the GFP status of the resulting offspring. Previous experimental tests showed that the reproductive performance of GFP(+) individuals is not different from those of GFP(–) individuals (Marie‐Orleach et al. 2014). In this study, we used two outbred cultures, the GFP(+) BAS1 culture (Marie‐Orleach et al. 2016; Vellnow et al. 2018) and the GFP(–) LS1 culture (Marie‐Orleach et al. 2013), that are expected to be genetically similar. This is because the BAS1 culture was established by introgression of the GFP marker into the LS1 culture (see Marie‐Orleach et al. 2016 for details on the establishment of the initial BAS1 culture; and Vellnow et al. 2018 for details on sorting out a karyotype polymorphism that initially existed in that culture, which could affect the penetrance of the GFP marker; see also below).

### EXPERIMENTAL SETUP

In this study, we aimed to (1) assess different components of a focal sperm donor's male fitness during different pre‐ and postcopulatory episodes of selection, and (2) determine the repeatability of individual success in these fitness components. The experimental setup built up upon a previous study (Marie‐Orleach et al. 2016). Here, we add novel features in the experimental setup (Fig. 1), intending to fill existing gaps in the literature on variance‐based sexual selection metrics. Specifically, we assessed the reproductive performance of focals in three different mating groups, which allowed us to assess the repeatability of individual success across these groups, and we nearly tripled the sample size compared to our earlier study, aimed at increasing the power and reducing the confidence intervals for previously obtained estimates. For logistical reasons, the replicates were divided into eight experimental batches that were treated sequentially, each three to six days apart, but the batches were otherwise exposed to standard conditions. For sake of clarity, in the following we set day 1 as the first day of each batch.

#### Rearing conditions

On day 1, we placed 100 and 500 adult individuals, respectively, from the GFP(+) and the GFP(–) culture into one and five Petri dishes (i.e., 100 worms per dish), and allowed them to lay eggs for 24 h to obtain offspring of similar age. The same parental worms were used for all eight batches. On day 6‐10 (depending on the batch), we collected the resulting offspring and placed them in 24‐well tissue culture plates (TPP AG, Switzerland) to create 20 biological replicates per batch. Each biological replicate included (i) one group composed of one GFP(+) focal and four GFP(–) partners (called the A groups), and (ii) three groups composed of four GFP(–) individuals (called the B, C, and D groups), which were to become the future partners of the focal in that replicate. All groups were moved to new wells with fresh algae every 6‐10 days (to ensure ad libitum food conditions and to avoid any sexual interactions with the offspring). Given that *M. lignano* reaches sexual maturity ∼18 days after egg laying (Schärer and Ladurner 2003), we kept the worms in these groups for long enough (see below) to allow them to copulate multiply with their group members, and to thus likely reach a steady state of sperm production, sperm donation, sperm receipt, and egg production before we assessed the pre‐ and postcopulatory components of their reproductive success.

#### Mating success

We estimated focal mating success on day 25‐30, depending on the batch (but variation across batches is probably not very important, because worms were expected to have reached a steady state; see also Results). For this, each focal was removed from its A group and placed in mating chambers together with the four individuals of its B group. To visually distinguish the focal from its partners during the mating trials, we placed all members of the A group into a well containing a blue food dye 24 h prior to the mating trial (0.25 mg/mL; E‐131; Werner Schweizer AG, Switzerland). This dyeing procedure has been shown not to affect sexual performance (Marie‐Orleach et al. 2013). The mating trials followed the mating chamber protocol described in detail elsewhere (Schärer et al. 2004). Here, the five worms were placed in an 8‐μL drop of artificial sea water between two microscope slides. We used a lateral illumination to make it easier to distinguish the blue‐dyed focal, and recorded at 1 frame/s for 3 h, using a digital video camera (DFK 41AF02, The Imaging Source Europe GmbH, Bremen, Germany) and the software BTV Pro 6.0b7 (http://www.bensoftware.com/). We included five such groups per mating chamber, for a total of four mating chambers per batch. The time‐lapse movies were later analyzed blindly with regard to replicate identity using the software KMPlayer (version 4.1.2.2). For each mating group, we counted the total number of copulations (total matings) and the number of copulations in which the focal was involved (focal matings). Immediately after the mating trials, we transferred the focal back into its A group, and isolated all four members of the B group to assess the sperm‐transfer success of the focal during the mating trial.

#### Sperm‐transfer success

Immediately after the mating trials, we estimated the proportion of sperm cells received from the focal by the four partners. This was done by following a protocol described in detail elsewhere (Janicke et al. 2011, 2013; Marie‐Orleach et al. 2014). Briefly, this protocol consists of anesthetizing and squeezing worms between two cover slips, and recording a movie while slowly focusing through the female antrum, first under bright field illumination and then under epifluorescence illumination at a 630× magnification. For this, we used a Leica DM2500 microscope (Leica Microsystems, Heerbrugg, Switzerland), an epifluorescence light source, a GFP filter cube (11513890, Leica Microsystems), and a digital microscope camera (Leica DFC360 FX, Leica Microsystems). The resulting antrum movies were analyzed later, blindly with regard to replicate identity, using KMPlayer. We assessed (i) the total number of sperm cells stored in the female antra across all four potential partners (total sperm), and (ii) the number of sperm that were GFP(+) (focal sperm). This procedure provides highly repeatable sperm counts (Marie‐Orleach et al. 2014). However, total sperm cannot be accurately estimated in worms that have one or several eggs in their female antrum while recording the movie (731 recipients out of 1800 in total), because this prevents reliable observation of GFP(–) sperm. In these cases, we used the observed average number of total sperm among those worms that could be estimated (i.e., 21 sperm cells), as previously done in Marie‐Orleach et al. (2016).

#### Reproductive success

Immediately after assessing sperm‐transfer success, partner individuals were again isolated to permit egg production for 12 days (a period during which most worms are expected to run out of received sperm to produce offspring; L. Marie‐Orleach, pers. obs.). We then counted the total number of offspring produced across all four potential partners (total offspring) and determined the number of these that were GFP(+) (focal offspring).

On the two subsequent days, we repeated all of the abovementioned steps, by placing each focal with the four individuals of their C and D groups, respectively. This repeated procedure allowed us to measure the same pre‐ and postcopulatory components of male reproductive success for each focal in three independent mating groups, sampled from the same pool of similarly aged outbred worms. In total, we observed 1350 h of mating interactions, during which we scored 26,203 copulations. We determined that the 1800 mating partners had received 37,938 sperm cells and produced 10,452 offspring.

#### Penetrance of the GFP marker

Finally, because the GFP marker may, in some cases, not be inherited by the offspring (Marie‐Orleach et al. 2014, 2016), we estimated the penetrance of the GFP marker for each GFP(+) focal, by pairing them with a virgin GFP(–) individual and assessing the GFP status of the resulting offspring (47.7 offspring screened per focal on average). This led to the exclusion of one focal that only produced 47% GFP(+) offspring (possibly a heterozygote for the GFP marker) and another one that did not produce any offspring at this stage of the experiment. The other focals produced either 100% (*n* = 135) or between 90% and 100% GFP(+) offspring (*n* = 23), which will not affect our reproductive success estimates much. Therefore, we did not correct for individual variation in the penetrance of the GFP marker. Note that in Marie‐Orleach et al. (2016), we did statistically account for this variation because it was substantially higher (i.e., the 52 focals produced either less than 90% [*n* = 21], between 90% and 100% [*n* = 10], or 100% [*n* = 21] GFP(+) offspring). Deviations from full inheritance of the GFP marker may be due to karyotype polymorphism observed in *M. lignano* (Zadesenets et al. 2016, 2017, 2020). But here, we used a GFP(+) BAS1 culture in which karyotype polymorphism and heterozygosity was deliberately reduced (Vellnow et al. 2018), which successfully led to much less variation in the inheritance of the GFP marker compared to the earlier study.

### DATA ANALYSIS

To estimate the repeatable opportunity for selection arising from each fitness component, we used a bootstrapping protocol in which we weighed the variance arising from each fitness component by the corresponding repeatability of the individual success. In the following, we first explain the decomposition of the variance observed in male reproductive success into the four multiplicative fitness components. Second, we explain how we estimate the repeatability of individual success along the same four fitness components. And third, we explain how we combine the variance and repeatability estimates.

#### Variance decomposition

We decomposed the variance observed in male reproductive success using an established variance decomposition approach (Arnold and Wade 1984; Collet et al. 2012; Pélissié et al. 2012, 2014; Rose et al. 2013; Janicke et al. 2015; Turnell and Shaw 2015; Marie‐Orleach et al. 2016) and, more specifically, following “model (2)” in Marie‐Orleach et al. (2016). For each replicate, we first summed across all three mating groups the number of total matings, focal matings, total sperm, focal sperm, total offspring, and focal offspring. Then we computed the four fitness components: total offspring (*F*), mating success (*MS*), sperm‐transfer efficiency (*STE*), and sperm fertilizing efficiency (*SFE*), as explained in Figure 1. That procedure allowed us to decompose male reproductive success (*mRS*) according to a deterministic multiplicative model, which is necessary for variance decomposition (Arnold and Wade 1984). Male reproductive success was decomposed as follows:

mRS=F×MS×STE×SFE.

For analysis, we transformed our fitness estimates to relative fitness estimates, which are hereafter denoted by an asterisk, by dividing each fitness component by its average (Jones 2009). This step makes the variance in relative male reproductive success equal to the sum of the variances in the relative fitness components and of twice their co‐variances when fitness data follow normal distributions (Arnold and Wade 1984). Thus, we decomposed the variance observed in male reproductive success (mRS*) into the four fitness components: partner fecundity (F*), mating success (MS*), sperm‐transfer efficiency (STE*), and sperm fertilizing efficiency (SFE*), and their covariances. We computed the 95% confidence intervals for each variance and covariance estimate by bootstrapping (10,000 iterations). Importantly, because STE* and SFE* were derived fitness components, spurious variance may arise due to sampling error and thus artificially inflate the variances observed in these fitness components. We accounted for this error variance by estimating the expected variance from a binomial sampling error, which we then subtracted from the observed variance to obtain unbiased variance estimates (for more details, see Pélissié et al. 2012 and Marie‐Orleach et al. 2016). We compared the variance arising from each of the four fitness components by using a pairwise signed difference test, as in Marie‐Orleach et al. (2016). In brief, we used a bootstrapping technique in which we assessed the difference of variance arising from two fitness components in each of 10,000 iterations. We then used the occurrence of positive and negative differences to derive a *P*‐value (two‐tailed test).

#### Repeatability of focal success

We computed the repeatability of male reproductive success and of each of the four fitness components across the three mating groups used in the experiment. For this, we first transformed our fitness measures estimated in each group as follows: √(*mRS*), √(*F* + 0.5), √*STE*, and log_10_(*SFE* + 1). These data transformations were done to provide satisfactory LMM diagnostic plots (“Normal Q‐Q” and “Residuals vs. Fitted” plots). Then, we relativized the data so that all fitness components have a mean of 1 (Jones 2009). Finally, we computed repeatability for each fitness component using the *rptR* R‐package (Stoffel et al. 2017), where a 95% confidence interval was estimated by bootstrapping (10,000 iterations). For all four fitness components, we assumed Gaussian data distributions because we wanted to assess the repeatability values for individual success across the three groups, whereas using binomial data distributions provides repeatability values at the observation level (i.e., each mating, each sperm, and each offspring).

We also tested if individual success in mRS* and all four fitness components changed consistently over the three mating groups (which might indicate that the worms were not in the steady state we hoped to achieve with our design) and over the eight experimental batches. For this, we fitted linear mixed models using the lme4 (Bates et al. 2015) and lmerTest (Kuznetsova et al. 2017) R‐packages to predict the fitness estimates with *mating group*, *batch*, and the *mating group* × *batch* interaction as fixed effects and individual ID as a random effect. We used the same data transformations as above.

#### Combining variance and repeatability estimates

Given that the repeatability estimates correspond to the proportion of total variance in the fitness estimates explained by focal ID, we can estimate the amount of variance observed in individual fitness that is repeatable over the three groups (i.e., the repeatable opportunity for selection) by combining the repeatability and variance estimates as follows:

$$I_{R}=\;R\times I,$$

where *I_R_* is the repeatable opportunity for selection, *R* is the repeatability for individual success over the three groups, and *I* is the standardized variance in individual success (i.e., the opportunity for selection). Note that the repeatable opportunity for selection was computed in each fitness component using total variance (i.e., without subtracting the binomial sampling error in STE* and SFE* to avoid a downward bias in the repeatable opportunity for selection). We estimated the repeatable opportunity for selection in each fitness component using a bootstrapping protocol with 10,000 iterations. Specifically, for each fitness component, we randomly sampled 150 replicates in our dataset (with replacement), on which we computed the product between the variance in all four fitness components, and the respective repeatability of all four fitness components over the three mating groups. Thus, we obtained the average and the 95% confidence intervals of the repeatable portions of variance for each of the four fitness components.

Finally, we statistically compared the repeatable opportunity for selection arising from the four fitness components by using a pairwise signed difference test. In brief, we computed the difference between the repeatable amounts of the variance found in two fitness components in a 10,000 iteration bootstrapping. We then used the occurrence of positive and negative differences to derive a *P*‐value (two‐tailed test).

#### Sample sizes

Our initial sample size was 160 replicates, but because we lost eight and two replicates due to developmental errors and the GFP penetrance assay, respectively, the final sample size was reduced to 150 replicates. Also, some of these replicates had missing values on specific fitness component for one or more mating groups. Missing data arose when fitness components could not be estimated. For example, sperm fertilizing efficiency could not be estimated when the focal did not successfully transfer sperm to its four partners. In addition, the recording of one mating movie failed. Hence, the final dataset was as follows: mRS* (three values, *n* = 150), F* (three values, *n* = 150), MS* (three values, *n* = 145; two values, *n* = 5), STE* (three values, *n* = 143; two values, *n* = 7), and SFE* (three values, *n* = 134; two values, *n* = 10; one value, *n* = 3; zero values, *n* = 3).

Statistical analyses were performed in R (version 3.6.3) (R Core Team 2020).

## Results

### OPPORTUNITY FOR SELECTION IN FITNESS COMPONENTS

**Figure 2:**
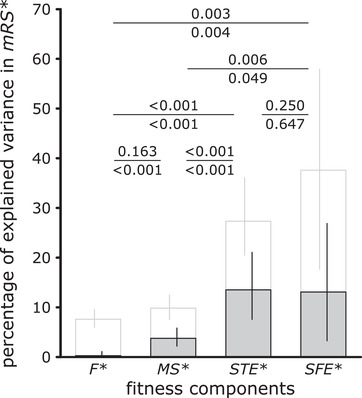
Decomposition of the variance observed in male reproductive success (mRS*) into four multiplicative fitness components, namely, partner fecundity (F*), mating success (MS*), sperm transfer‐efficiency (STE*), and sperm fertilizing efficiency (SFE*). All fitness estimates are relativized (so that the means equal 1), which is denoted by the asterisks. The white bars show the total variance arising from each of the four fitness components, whereas the gray portions of these bars show the amount that is repeatable—the repeatable opportunity for selection—estimated by taking into account the repeatability of individual fitness components across the three independent mating groups. The error bars represent 95% confidence intervals estimated via bootstrapping. The *P*‐values test for the pairwise comparisons of variance component estimates (above the bar) and the repeatable component of the variance estimates (below the bar) estimated via bootstrapping (see Methods for details and Fig. S1 for a more complete variance decomposition).

Most of the variance observed in male reproductive success arose from the two postcopulatory fitness components (Table 1; Fig. 2, white bars). We accounted for the variance due to sampling error (i.e., sampling a finite number of sperm and offspring to assess the proportion of focal sperm and focal offspring, respectively) by computing the amount of variance expected from a binomial sampling error in STE* (1% [0‐2%]) and in SFE* (27% [12‐55%]), which we subtracted from the corresponding observed variance (see the Methods for details). The covariances between the different fitness components explained −10% of variance in total, but, as in Marie‐Orleach et al. (2016), none of the six covariances significantly differed from zero (see Fig. S1), so we do not further discuss this outcome here. Finally, as in Marie‐Orleach et al. (2016), we found less variance in mRS* (0.68) than what our model predicted (0.95), which is likely due to the somewhat skewed distributions of our fitness components.

**Table 1:** Opportunity for selection (*I*), repeatability (*R*), and repeatable opportunity for selection (*I_R_*), calculated for each of the four fitness components, partner fecundity (F*), mating success (MS*), sperm‐transfer efficiency (STE*), and sperm fertilizing efficiency (SFE*). *I_R_* is estimated as *I* × *R* (see Methods for details). *I* and *I_R_* are expressed as a percentage of explained variance in male reproductive success. The ranges in the brackets represent the 95% confidence interval obtained by bootstrapping. The binomial sampling error estimated in STE* and SFE* was subtracted from the respective *I*. *R* was estimated after applying the following data transformations: √(*F* + 0.5), √*STE*, and log_10_(*SFE* + 1). See Figure 2 for pairwise comparisons. See Methods and Results for details

Fitness components	Opportunity for selection(*I*)	Repeatability(*R*)	Repeatable opportunity for selection(*I_R_*)
F*	8% [5‐11%]	0.03 [0.00‐0.13]	0% [0‐1%]
MS*	10% [7‐13%]	0.39 [0.28‐0.48]	4% [2‐6%]
STE*	27% [20‐37%]	0.47 [0.37‐0.56]	13% [8‐21%]
SFE*	37% [19‐54%]	0.21 [0.10‐0.32]	13% [3‐27%]

### REPEATABILITY OF FOCAL SUCCESS ACROSS GROUPS

**Figure 3:**
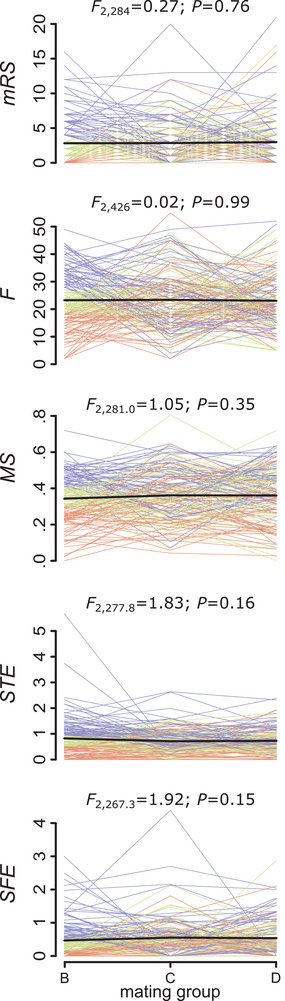
Individual success in male reproductive success (*mRS*) and the four multiplicative fitness components over the three mating groups. The fitness components are partner fecundity (*F*), mating success (*MS*), sperm‐transfer efficiency (*STE*), and sperm fertilizing efficiency (*SFE*). Each thin line corresponds to a focal and the lines are colored according to the focal's success in mating group B (bottom third in orange, middle third in green, top third in blue) to visualize that the success is more consistent across the groups for some fitness components (i.e., when the colors remain stratified as, e.g., for *STE*) than for others (i.e., when the colors appear mixed as, e.g., for *F*). The thick black line represents the average over all focals. Summary statistics above each panel are for the effect of mating group on each fitness estimate (see Methods for details and Table S1 for full summaries).

Male reproductive success was significantly repeatable (mean R [95% CI] = 0.33 [0.22‐0.43]), meaning that some focals sired consistently more (or consistently fewer) offspring across the three mating groups than other focals did (see also Fig. 3). In contrast, partner fecundity, F*, showed a low and nonsignificant repeatability (Table 1), which means that the number of offspring produced by the partners did not depend on the focals. The repeatability estimates of the other three fitness components were all significant (Table 1). Specifically, the precopulatory performance of the focals was, to some extent, consistent over the three groups, as we found a significant repeatability for mating success, MS*, whereas among the postcopulatory components sperm‐transfer efficiency, STE*, seemed to be more consistent than sperm fertilizing efficiency, SFE*, which suggest that the other sperm donors, the sperm recipients, and/or stochastic events had less influence on STE* than on SFE*.

It is important to consider that the repeatability estimates could be biased if the fitness estimates varied consistently across the three mating groups or the eight experimental batches that we ran (see Methods). To avoid such potential biases, the focals were kept under standardized conditions in groups of five worms prior to the mating trials and throughout the experiment, allowing them to reach a steady state (see Fig. 1 and Methods for details). We found that none of the fitness estimates showed a significant overall trend across the three mating groups (Fig. 3; Table S1), suggesting that the focals were indeed in a steady state over the three mating groups. However, we detected significant differences in F* across the eight experimental batches (*P* < 0.001; Table S1), which could have inflated our repeatability estimate of this fitness component (but we did not try to account for this batch effect statistically given that F* was anyway not significantly repeatable). Moreover, this batch effect on F* may explain why mRS* also differed across batches (*P* < 0.031; Table S1). But importantly, we did not find differences in MS*, STE*, and SFE* across the batches (Table S1), suggesting that the significant repeatabilities in these fitness components did not stem from differences across batches.

### REPEATABLE OPPORTUNITY FOR SELECTION IN FITNESS COMPONENTS

The repeatable opportunity for selection found in the precopulatory fitness component, MS*, was more than three times lower than what we found in both postcopulatory fitness components, STE* and SFE* (Table 1; Fig. 2, gray bars). This result clearly suggests that sexual selection is stronger in the postcopulatory compared to precopulatory episodes of selection in *M. lignano*. Moreover, because F* had a very low repeatability, the repeatable portion of variance was not significantly different from zero, and was thus significantly lower than the one arising from all other three fitness components. The repeatable amounts arising from STE* and SFE* were not significantly different from each other (Fig. 2).

## Discussion

Our results support previous evidence that postcopulatory fitness components can exhibit large opportunities for selection (Collet et al. 2012; Pélissié et al. 2012, 2014; Rose et al. 2013; Devigili et al. 2015; Janicke et al. 2015; Turnell and Shaw 2015; Marie‐Orleach et al. 2016), and thereby improve our knowledge about the relative strength of pre‐ versus postcopulatory selection. By measuring individual success over multiple independent mating groups, we could measure the repeatable opportunity for selection arising from pre‐ and postcopulatory fitness components in the simultaneously hermaphroditic flatworm *M. lignano*, and thereby obtain better estimates of the relative strength of pre‐ and postcopulatory sexual selection compared to previous studies. Specifically, our results suggest that the two postcopulatory fitness components—namely, sperm‐transfer efficiency (*STE*) and sperm fertilizing efficiency (*SFE*)—experience stronger selection than the precopulatory component, mating success (*MS*), which is also consistent with *M. lignano* having many traits that appear to be involved in postcopulatory sexual selection and sexual conflicts, as we discuss in more detail below.

The decomposition of the variance in male reproductive success into pre‐ and postcopulatory episodes of selection has now been achieved in several studies (Collet et al. 2012; Pélissié et al. 2012, 2014; Rose et al. 2013; Devigili et al. 2015; Janicke et al. 2015; Turnell and Shaw 2015; Marie‐Orleach et al. 2016), and a recent review suggested that postcopulatory episodes of selection often explain larger proportions of variance in male reproductive success than precopulatory components (Evans and Garcia‐Gonzalez 2016). In most of these studies, postcopulatory sexual selection was, however, assessed through a single fitness component, whereas in our study—because our model organism permits *in vivo* sperm tracking*—*we could quantify the outcome of postcopulatory episodes of selection through two fitness components, sperm‐transfer efficiency (*STE*), and sperm fertilizing efficiency (*SFE*). Our results show that both of these fitness components are of comparable importance in determining male fitness. Given that many copulations occurred during the mating trials, sperm‐transfer efficiency likely captures the ability of the focals to transfer sperm to partners, but probably also to some degree the ability of the focals’ sperm to resist being displaced from the female antrum (either by subsequent sperm donors, actively by the sperm recipients themselves, or passively, such as during egg laying). Sperm displacement is likely also captured by the sperm fertilizing efficiency fitness component, but only displacement by the sperm recipient and passive sperm loss. Moreover, sperm fertilizing efficiency may also capture the ability of the focal sperm to compete against rival sperm once in storage, and to efficiently fertilize the eggs as they become available. Thus, it seems more likely that individual success in these fitness components is due to several traits, or combinations of traits, rather than single traits.

These results concur with the multiple behavioral and morphological postcopulatory traits found in this species. After copulation, worms often bend down on themselves and place their pharynx over the opening of their own female antrum, and appear to suck for about 5 s (Schärer et al. 2004). This intriguing behavior is thought to be a female resistance trait involved in sexual conflicts over the fate of the received sperm (Vizoso et al. 2010; Schärer et al. 2011; Marie‐Orleach et al. 2013). Recent evidence shows that the seminal fluid of these worms contains proteins that reduce the propensity of the partners to perform this suck behavior (Patlar et al. 2020), and these seminal fluids therefore represent a male persistence trait involved in sexual conflicts between mating partners over the fate of the donated sperm. Moreover, the morphology of the sperm cells may also reflect additional male persistence traits to postcopulatory sexual selection and conflict. Sperm cells are often seen anchored in the epithelium of the female antrum with an anterior sperm feeler and they have two stiff lateral sperm bristles (Vizoso et al. 2010), which may help sperm cells to avoid being displaced from the female antrum (e.g., during the recipient's suck behavior, during egg laying, or during following copulations) and thereby increase the chance of fertilization. The evolution of these postcopulatory traits indicates that postcopulatory sexual selection and sexual conflict are key components of selection in this species (Schärer et al. 2011).

The major novelty of our study is that we determined the repeatability of focal success, which allowed us to estimate the repeatable opportunity for selection arising from each fitness component. We find that partner fecundity is not repeatable across mating groups (i.e., the fecundity of the partners was probably not influenced by the focal identity), leading to a repeatable opportunity for selection in partner fecundity that is not different from zero. This outcome may indicate that there is no ongoing selection on traits, such as for instance seminal‐fluid proteins, that affect partner fecundity in *M. lignano* (but see below). In contrast, we found that mating success, sperm‐transfer efficiency, and sperm fertilizing efficiency were all repeatable to some degree, with sperm‐transfer efficiency having the highest repeatability. By combining these repeatability estimates with our variance decomposition, we find higher repeatable opportunity for selection arising from both sperm‐transfer efficiency and sperm fertilizing efficiency compared to mating success. This result suggests that postcopulatory sexual selection is stronger than precopulatory sexual selection in *M. lignano*, at least in the experimental paradigm used here.

The distinction between the variance due to deterministic factors versus stochastic events in our study directly addresses previous criticisms of the variance‐based approach for measuring sexual selection. Several authors have argued that the variance‐based approach may be misleading because variance may arise due to either deterministic factors or stochastic events (Klug et al. 2010; Jennions et al. 2012), and we broadly agree with this critique. Moreover, we think that this issue may be especially problematic in studies that compare variances across fitness components, because the share of variance due to deterministic factors versus stochastic events may well differ across fitness components, as we show here. For instance, some studies have accounted for the inferred (Pischedda and Rice 2012) or observed (Pélissié et al. 2014) mating order of sperm donors when sperm recipients have mated multiply, and found that mating order explained a large proportion of the variance in male reproductive success. In species with either first‐ or last‐male sperm precedence, such an outcome is actually expected. However, it is unknown in these studies whether the mating order that was accounted for actually had a deterministic source. Being the first or the last sperm donor in a mating trial may either be random with regard to the identity or morphology of the donor (in which case the mating order has a stochastic source), and/or it could involve effects where a sperm donor can affect the remating rate of the sperm recipients—for example, via a seminal fluid—thus increasing the likelihood of this sperm donor being the last to mate (in which case the mating order may have a deterministic postcopulatory source). Because selection can only occur in the later scenario (e.g., on components of seminal fluid function), it is critical to assess the relative contribution of deterministic factors in the total variance by testing the repeatability of individual success.

Another interesting consideration is that the consistency of individual success across multiple groups may be affected by stochastic variation in the group composition. For instance, if individual success can be strongly affected by the genotypes of the sperm recipients and/or competing sperm donors, and if the different groups differ in their genotypic composition, then one would expect to observe lower repeatability in individual success despite selection still occurring (e.g., via genotype × genotype interactions). Mating latency and the propensity of the postcopulatory suck behavior have been shown to depend on the genotype of the mating partner in *M. lignano* (Marie‐Orleach et al. 2017), but neither mating success nor sperm‐transfer success seems to be influenced by partner genotype (Nikolas Vellnow, pers. obs.). For instance, the lack of repeatability in partner fecundity may either mean that there is no ongoing selection on traits manipulating partner fecundity, or that the effectiveness of the traits manipulating partner fecundity strongly depends on the genotype of the sperm recipient. A recent study has identified several seminal‐fluid transcripts affecting partner fecundity (Weber et al. 2019), but it is unknown whether there is standing genetic variation in genes underlying that trait on which selection could act.

Overall, our results support the theoretical argument that sexual selection is shifted toward the postcopulatory arena in simultaneous hermaphrodites (Charnov 1979; Michiels 1998; Schärer and Pen 2013). This is thought to be the case because, in contrast to gonochorists, when two simultaneous hermaphrodites meet, individuals may share a preference for assuming only one of the mating roles, which may often be the male role (Charnov 1979; Schärer et al. 2014), leading to sexual conflict. This conflict may arguably be resolved through reciprocal copulation, which allows both individuals to assume their preferred role, sperm donation, at the expense of also assuming the less preferred role, sperm receipt (Charnov 1979). If all individuals are eager to copulate, then male reproductive success is no longer strongly limited by the number of matings achieved (a largely precopulatory matter) but instead by the ability to convert any matings into successful fertilization of the eggs of the mating partners (involving largely postcopulatory processes). Our results, alongside other studies (Koene and Schulenburg 2005; Schärer et al. 2011; Nakadera et al. 2014; Patlar et al. 2020), clearly support the view that postcopulatory sexual selection and sexual conflict is indeed a critical component of sexual selection in simultaneous hermaphrodites. Ultimately, additional studies comparing the strength of pre‐ and postcopulatory sexual selection in hermaphroditic and gonochoristic species are needed to draw a more general conclusion on whether sexual selection is indeed shifted toward the postcopulatory arena in hermaphroditic species compared to gonochoristic species.

## CONCLUSIONS

We performed mating observations, *in vivo* sperm tracking, and paternity analyses to quantify the strength of selection on pre‐ and postcopulatory fitness components in the flatworm *M. lignano*. Our results suggest that postcopulatory episodes of selection experience overall stronger selection, because we found a significantly higher repeatable opportunity for selection (*I_R_*) in sperm‐transfer efficiency and sperm fertilizing efficiency compared to mating success. These results represent clear evidence that more intense selection results from postcopulatory episodes of selection in *M. lignano*. In general, assessing the repeatability (*R*) of fitness components over multiple independent groups allows to estimate how much of the opportunity for selection (*I*) might be due to deterministic rather than stochastic factors, which is critical to predict the response to selection and subsequent evolution. The next step toward this aim is assessing the genetic and phenotypic determinants of individual success on different fitness components.

## CONFLICT OF INTEREST

The authors declare no conflict of interest.

## AUTHOR CONTRIBUTIONS

LMO and LS conceived the study and designed the experiment. LMO and NV performed the experiment. LMO collected and analyzed the data. LMO and LS wrote the manuscript. All authors agreed on the final version.

## DATA ARCHIVING

The dataset and the R‐scripts are in the supporting information and on the Dryad data repository (https://doi.org/10.5061/dryad.9w0vt4bdg). The mating movies and the antrum movies are deposited on Zenodo (https://doi.org/10.5281/zenodo.3952492).

Associate Editor: R. Snook

## Supporting information


**Figure S1**. Decomposition of variance observed in male reproductive success (mRS*) along four fitness components (red), their covariances (blue), and the binomial sampling errors (yellow).

**Table S1**. Summary statistics of the linear mixed models testing for the effect of the mating group and batch on male reproductive success (mRS*) and the four fitness components, namely partner fecundity (F*), mating success (MS*), sperm‐transfer efficiency (STE*), and sperm fertilizing efficiency (SFE*).

Supplementary Material
