# Supplementary material for: The repeatable opportunity for selection differs between pre‐ and postcopulatory fitness components
Source: Evol Lett. 2020 Dec 25;5(1):101–14. doi: 10.1002/evl3.210 (PMC7857279; doi:10.1002/evl3.210)
Supplement: Supplementary file 1 — Figure S1. Decomposition of variance observed in male reproductive success (mRS*) along four fitness components (red), their covariances (blue), and the binomial sampling errors (yellow). [file EVL3-5-101-s001.pdf]

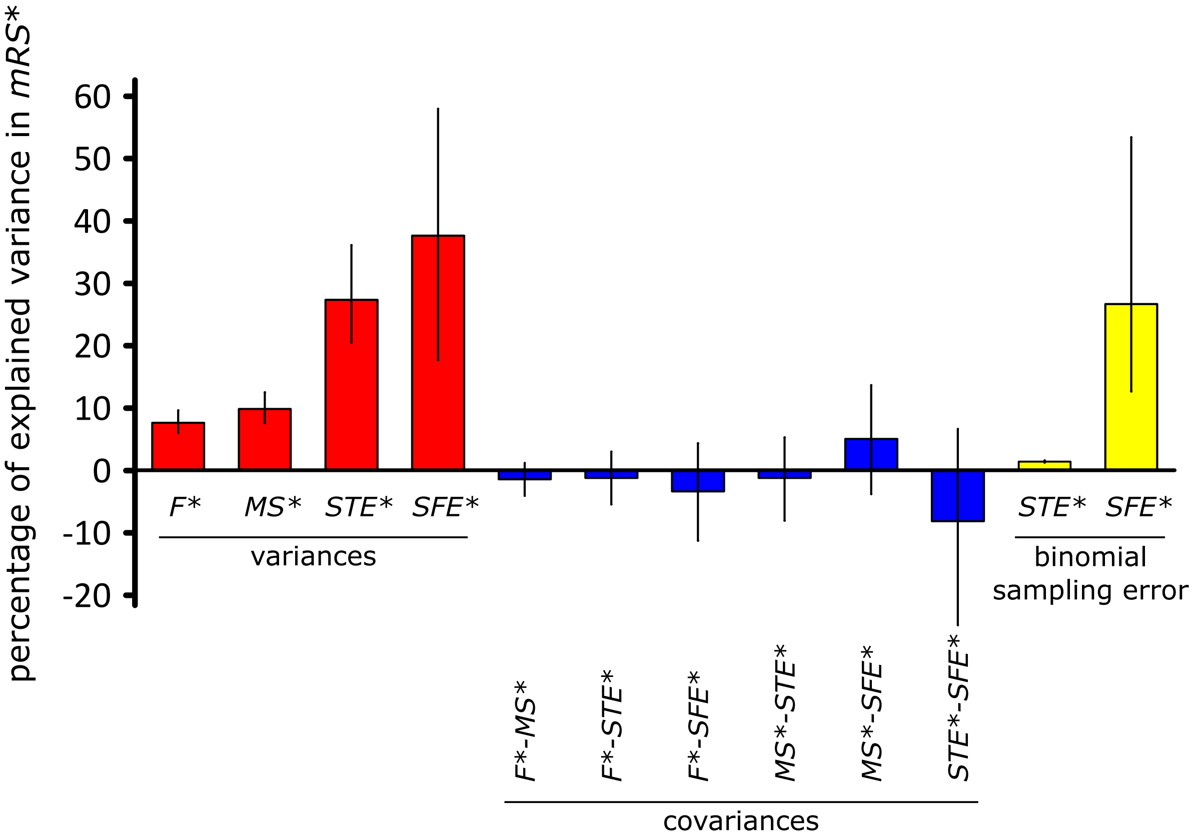

Figure S1. Decomposition of variance observed in male reproductive success (*mRS) along four fitness components (red), their covariances (blue), and the binomial sampling errors (yellow).** Fitness components are partner fecundity (*F**), mating success (*MS**), sperm-transfer efficiency (*STE**) and sperm fertilising efficiency (*SFE**). Error bars represent the bootstrapped 95% percentile confidence intervals. See Methods and Results for details.
