# Supplementary material for: The repeatable opportunity for selection differs between pre‐ and postcopulatory fitness components
Source: Evol Lett. 2020 Dec 25;5(1):101–14. doi: 10.1002/evl3.210 (PMC7857279; doi:10.1002/evl3.210)
Supplement: Supplementary file 2 — Table S1. Summary statistics of the linear mixed models testing for the effect of the mating group and batch on male reproductive success (mRS*) and the four fitness components, namely partner fecundity (F*), mating success (MS*), sperm‐transfer efficiency (STE*), and sperm fertilizing efficiency (SFE*). [file EVL3-5-101-s002.pdf]

Table S1. Summary statistics of the linear mixed models testing for the effect of the mating group and batch on male reproductive success (*mRS) and the four fitness components, namely partner fecundity (*F**), mating success (*MS**), sperm-transfer efficiency (*STE**), and sperm fertilising efficiency (*SFE**).** The response variables were first transformed as follow: √(*mRS*), √(*F*+0.5), √*STE*, and $\log_{10}$(*SFE+1*), and then relativized. All models included focal ID as a random effect. See Methods and Results for details.

Response	Source	*df*	*F* value	*P* value
*mRS**	mating group	2, 284	0.27	0.763
	batch	7, 142	2.28	0.031
	mating group x batch	14, 284	0.95	0.507
*F**	mating group	2, 426	0.02	0.985
	batch	7, 426	6.22	<0.001
	mating group x batch	14, 426	1.25	0.236
*MS**	mating group	2, 281.0	1.05	0.351
	batch	7, 142.5	1.90	0.073
	mating group x batch	14, 280.8	0.74	0.738
*STE**	mating group	2, 277.8	1.83	0.162
	batch	7, 141.1	0.94	0.480
	mating group x batch	14, 277.6	1.37	0.165
*SFE**	mating group	2 , 267.3	1.92	0.148
	batch	7, 136.7	0.78	0.602
	mating group x batch	14, 267.0	0.82	0.644
